# Activation of the Canonical Wnt/β-Catenin Pathway in ATF3-Induced Mammary Tumors

**DOI:** 10.1371/journal.pone.0016515

**Published:** 2011-01-31

**Authors:** Leqin Yan, Luis Della Coletta, K. Leslie Powell, Jianjun Shen, Howard Thames, C. Marcelo Aldaz, Michael C. MacLeod

**Affiliations:** 1 Department of Molecular Carcinogenesis, The University of Texas MD Anderson Cancer Center, Smithville, Texas, United States of America; 2 Department of Biomathematics, The University of Texas MD Anderson Cancer Center, Houston, Texas, United States of America; SanfordBurnham Medical Research Institute, United States of America

## Abstract

Female transgenic mice that constitutively overexpress the transcription factor ATF3 in the basal epithelium of the mammary gland develop mammary carcinomas with high frequency, but only if allowed to mate and raise pups early in life. This transgenic mouse model system reproduces some features of human breast cancer in that about 20% of human breast tumor specimens exhibit overexpression of ATF3 in the tumor cells. The ATF3-induced mouse tumors are phenotypically similar to mammary tumors induced by overexpression of activating Wnt/β-catenin pathway genes. We now show that the Wnt/β-catenin pathway is indeed activated in ATF3-induced tumors. β-catenin is transcriptionally up-regulated in the tumors, and high levels of nuclear β-catenin are seen in tumor cells. A reporter gene for Wnt/β-catenin pathway activity, TOPGAL, is up-regulated in the tumors and several downstream targets of Wnt signaling, including *Ccnd1*, *Jun*, *Axin2* and *Dkk4*, are also expressed at higher levels in ATF3-induced tumors compared to mammary glands of transgenic females. Several positive-acting ligands for this pathway, including *Wnt3*, *Wnt3a*, *Wnt7b*, and *Wnt5a*, are significantly overexpressed in tumor tissue, and mRNA for *Wnt3* is about 5-fold more abundant in transgenic mammary tissue than in non-transgenic mammary tissue. Two known transcriptional targets of ATF3, *Snai1 and Snai2*, are also overexpressed in the tumors, and Snail and Slug proteins are found to be located primarily in the nuclei of tumor cells. *In vitro* knockdown of *Atf3* expression results in significant decreases in expression of *Wnt7b, Tcf7, Snai2 and Jun*, suggesting that these genes may be direct transcriptional targets of ATF3 protein. By chromatin immunoprecipitation analysis, both ATF3 and JUN proteins appear to bind to a particular subclass of AP-1 sites upstream of the transcriptional start sites of each of these genes.

## Introduction

The bZip transcription factor, ATF3, has for some time been known to have the potential to both activate and repress transcription in a context-dependent manner [1]–[5]. ATF3 homodimers appear to be repressors [5], [6], whereas heterodimers with Jun or JunB activate gene expression [3]. Although ATF3 expression has clearly been associated with the DNA damage response and responses to other cellular stressors [2], [7]–[10], its exact role in these processes remains unclear. In some systems, ATF3 appears to function in tumor suppressive pathways [11], [12], and in particular has recently been associated with induction of apoptosis after treatments that induce lethal levels of DNA damage [13].

On the other hand, ATF3 has been shown to be growth stimulatory or anti-apoptotic in several systems [1], [14]–[19]. Several recent studies implicate ATF3 in tumorigenesis. In a transgenic mouse model in which human ATF3 is expressed from the bovine cytokeratin 5 promoter (BK5.ATF3), we reported spontaneous development of oral tumors, including about a 70% incidence of squamous cell carcinomas at 16 months of age [20]. We recently demonstrated that in this same model, parous female transgenic mice develop mammary tumors with squamous differentiation in the first year of life with an incidence of about 67% [21]. Although the transgene is expressed in the basal, myoepithelial compartment of the mammary gland throughout development, mammary tumors do not develop in virgin, transgenic females. Hai and colleagues have demonstrated overexpression of the ATF3 protein in a majority of human breast cancers [19], and we have found by immunohistochemistry (IHC) that in about 20% of human breast cancers ATF3 is found to be localized to the nuclei of the tumor cells; in the remaining ATF3-positive breast cancers, expression is primarily seen in stromal elements [21].

Phenotypically, mammary tumors induced in the BK5.ATF3 model resemble tumors and dysplastic lesions that have been reported in several transgenic models in which components of the Wnt/β-catenin pathway are overexpressed and the pathway is thereby constitutively activated [22]–[28]. For example, mammary tumors that arise in mice expressing a stabilized form of β-catenin from the cytokeratin 14 promoter express differentiation markers characteristic of both mammary epithelium, including cytokeratin 8, and epidermis, including cytokeratin 10 [28]. We have found aberrant expression of cytokeratins 5 and 8 in most tumor cells in the BK5.ATF3 model [21], as well as supra-basal expression of cytokeratins 6 and 10 and several cytokeratins that are characteristic of the inner root sheath of hair follicles. Interestingly, nuclear IHC staining for the ATF3 transgene is confined to the basal cell layer in the tumors [21]. In both types of models, squamous metaplastic histopathology is seen, with the tendency to form cyst-like structures with a core of keratinaceous material and cell debris, surrounded by a multi-layered epithelium that exhibits several features of squamous differentiation. These phenotypic similarities suggested the possibility that the Wnt/β-catenin pathway is somehow activated in ATF3-induced mammary tumors, or alternatively that ATF3 is a downstream effector of Wnt/β-catenin signaling. However, functional links between ATF3 and Wnt/β-catenin signaling have not been described in the literature.

The Wnt/β-catenin pathway is well known for its involvement in colon carcinogenesis [29]. About 85% of both familial and sporadic colon cancers involve mutations in the *APC* gene that lead to activation of the Wnt/β-catenin pathway [30]. Wnt/β-catenin signaling is absolutely required for mammary gland development, and acts at several critical time periods during pre- and post-natal development. Over the past decade, several epigenetic abnormalities in Wnt pathway genes have also been identified in human breast cancer. Promoter methylation of the APC gene has been found in about 40% of breast cancer cases [31], [32], and high levels of promoter methylation for several Wnt-inhibitory genes in the SFRP and DKK families in breast cancer have also been reported [33], [34].

The canonical pathway of Wnt/β-catenin signaling [35] begins with the interaction of an extracellular Wnt family protein with a transmembrane receptor of the Fz family; each of these gene families have more than a dozen members in mouse and human. This triggers formation of a complex with a second membrane co-receptor, Lrp5 or Lrp6, phosphorylation of both receptors, and binding of two cytoplasmic proteins, Disheveled and Axin, to the complex. In the unstimulated cell, cytoplasmic β-catenin associates with a so-called destruction complex, containing the proteins Axin, APC and Gsk3. In this complex, β-catenin is specifically phosphorylated by the kinase activity of Gsk3, which marks it for subsequent ubiquitylation and degradation by the proteasome. Following Wnt binding to the receptor, the destruction complex becomes tethered to the membrane ligand/receptor complex through Axin and loses its affinity for β-catenin, which then accumulates in the cytoplasm and is transported to the nucleus. Transcription factors of the Tcf/Lef1 family in the nucleus are then converted from repressors to activators by replacement of a repressive partner, Groucho, with the activating partner, β-catenin, thus activating transcription of an array of genes that constitute the downstream mediators of the pathway. In many systems, the pathway can be activated by adding exogenous Wnt protein, or by inhibiting the activity of Gsk3.

In the present study, we show that the Wnt/β-catenin pathway is functionally activated in mammary tumors induced in the BK5.ATF3 model. Initial probing of possible molecular links between ATF3 expression and Wnt/β-catenin identified robust up-regulation in ATF3-induced tumors of *Wnt3*, *Wnt3a*, *Wnt10b* and *Wnt7b*, several activating ligands for the canonical pathway.

## Results

### β-catenin is overexpressed in and localizes to the nuclei of BK5.ATF3 tumor cells

**Figure 1:**
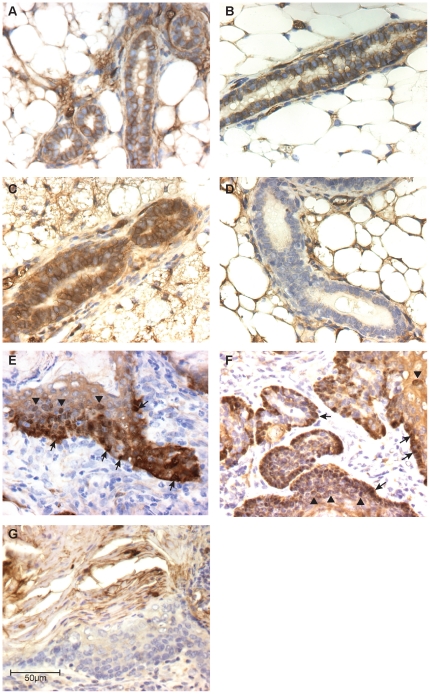
IHC analysis of β-catenin in ATF3-induced mammary tumors. β-catenin expression was assessed by immunohistochemistry in normal mammary glands of: **A**, young (18 weeks), non-parous BK5.ATF3 females; **B**, young (18 weeks), non-parous non-transgenic females; or **C,D**, older parous non-transgenic females; in panel **D**, non-specific rabbit IgG was substituted for the anti- β-catenin antibody as a negative control. Mammary tumors derived from parous BK5.ATF3 females were analyzed with the anti- β-catenin antibody (panels **E, F**), or with the IgG control (panel **G**). Arrows in panels **E** and **F** indicate typical nuclear staining of basal tumor cells, and arrowheads indicate supra-basal nuclei. Scale bar in **G** = 50 µm, applies to all panels.

In a previous study, similarities were noted between the histopathology of ATF3-induced mammary tumors and lesions described in several transgenic models in which the Wnt/β-catenin pathway is upregulated. Notably, expression of a stabilized form of β-catenin in basal cells from the Krt14 promoter resulted in squamous metaplastic lesions and carcinomas, similar to the pathology seen in BK5.ATF3 mammary tumors [28]; similarities in aberrant patterns of cytokeratin expression were also seen between these two models. However, there is no precedent in the literature for an effect of ATF3 on the Wnt/β-catenin pathway. To determine whether Wnt/β-catenin signaling is affected in the BK5.ATF3 mammary tumorigenesis model, we first used IHC to analyze the distribution of β-catenin in mammary glands and in ATF3-induced tumors. In the absence of Wnt/β-catenin pathway activation, β-catenin is important in maintaining epithelial intercellular communication [35] and is therefore found in normal mammary duct epithelial cells, typically showing a diffuse cytoplasmic distribution or localization to the basolateral plasma membrane in glands from either BK5.ATF3 transgenic (Figure 1A) or non-transgenic (Figure 1B) animals. Even in glands from older, parous females, where β-catenin staining was heavier (Figure 1C) normal ducts did not exhibit evidence of nuclear localization of β-catenin. In control sections with normal rabbit IgG substituted for the anti-β-catenin antibody, epithelial cells did not stain appreciably for β-catenin (Figure 1D). Nuclear localization of β-catenin is characteristic of canonical Wnt β-catenin pathway activation [35]. In ATF3-induced mammary tumors (Figure 1E,F), overall expression of β-catenin appeared to be stronger than in normal tissue, and clear localization to the nuclei of tumor cells was widespread. Nuclear β-catenin was seen extensively in the basal layers of the tumors (arrows, Figure 1E,F), but was also seen in supra-basal cells (arrowheads, Figure 1E,F). In ten independent tumors examined in detail, an average of 43% of tumor cells exhibited clear nuclear expression of β-catenin. Nuclear labeling of tumor cells was abolished when normal rabbit IgG was substituted for the specific β-catenin antibody (Figure 1G).

**Figure 2:**
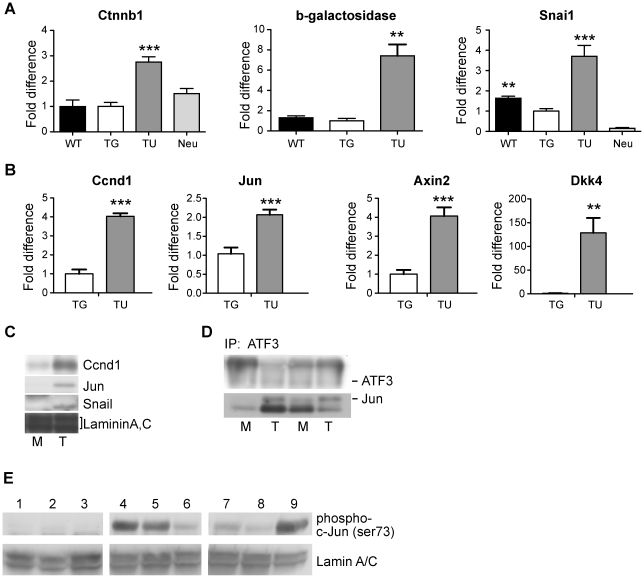
Expression analysis of Wnt/β-catenin pathway genes. Gene expression analysis was performed as described in Materials and Methods by quantitative real-time PCR for: **A**, *Ctnnb1*, bacterial β-galactosidase, *Snai1*; and **B**, *Ccnd1*, *Jun*, *Axin2*, and *Dkk4*. WT, mammary glands from non-transgenic mice; TG, mammary glands from BK5.ATF3 mice; TU, mammary tumors from BK5. ATF3 mice; Neu, mammary tumors from MMTV.neu mice. The mean and standard deviation of relative expression levels for at least 5 individual mice are shown; for all genes the mean expression level obtained with BK5.ATF3 mammary glands was set to 1.0. The statistical significance of differences between mean expression levels, calculated relative to BK5.ATF3 mammary glands, is indicated over the bars of the histograms: *, p<0.05; **, p<0.01; ***, p<0.001. **C**, immunoblots from a representative BK5.ATF3 mammary gland extract (M) and a mammary tumor (T) are shown for Cyclin D1, Jun and Snail; laminin A,C is shown as a protein loading control. **D**, extracts from paired mammary gland and tumor samples were immunoprecipitated with antibody to ATF3, then electrophoresed and immunoblotted for ATF3 and Jun. **E**, immunoblots of nuclear extracts from non-transgenic mammary glands (lanes 1–3), BK5.ATF3 transgenic mammary glands (lanes 4–6) or BK5.ATF3 mammary tumors (lanes 7–9) were prepared with a phospho-serine 73-Jun specific antibody; all lanes are from a single representative gel.

To confirm this finding, mRNA was prepared from normal mammary glands of young adult virgin females, ATF3-induced mammary tumors, and MMTV.neu-induced mammary tumors, and analyzed by quantitative polymerase chain reaction (qPCR). β-catenin mRNA was detected at similar levels in normal mammary tissue from non-transgenic mice and BK5.ATF3 heterozygous mice (Figure 2A). Seven independent ATF3-induced tumors exhibited an average of about three-fold higher levels of β-catenin mRNA as compared to normal mammary glands from BK5.ATF3 animals (t-test, p<.001). As expected, MMTV.neu induced tumors [36] had no such increase in β-catenin mRNA (p>.05).

### Downstream Wnt/β-catenin target genes are up-regulated in BK5.ATF3 tumors

To determine whether the nuclear localization of β-catenin correlated with activation of the Wnt/β-catenin pathway, we utilized a reporter strain of mice, TOPGAL [37], in which Wnt/β-catenin signaling drives the expression of a bacterial β-galactosidase gene in a non-tissue-specific manner. Doubly transgenic BK5.ATF3^Tg/0^;TOPGAL^Tg/0^ female mice, hemizygous for both transgenes, were allowed to raise two litters of pups, and then monitored for mammary tumor formation until 16 months of age. As expected, about two-thirds of these mice developed mammary tumors between the ages of 6 and 12 months. Indeed, the survival of these mice (Figure S1A) was virtually identical to that reported for singly transgenic BK5.ATF3 mice, and the histopathology of the resulting tumors (Figure S1B) was also unchanged by the TOPGAL transgene. By qPCR, expression of the β-galactosidase transgene was increased an average of about 7-fold in tumors produced in doubly transgenic mice compared to normal mammary glands of TOPGAL mice (Figure 2A; p<0.01). There was no significant difference in β-galactosidase expression between normal mammary glands of TOPGAL mice that did or did not carry the BK5.ATF3 transgene (p>0.05). These findings strongly indicate that the Wnt/β-catenin pathway is activated in these tumors, but not in normal mammary glands of BK5.ATF3 transgenic mice.

**Figure 3:**
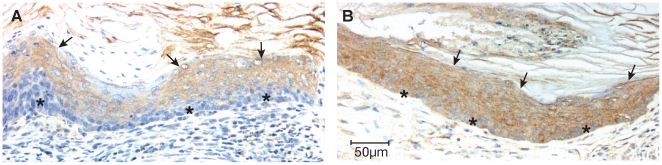
TOPGAL is expressed in ATF3-induced tumors. Immunohistochemistry was used to analyze expression of bacterial β-galactosidase in mammary tumors from mice of genotype: **A**, BK5.ATF3^Tg/0^;TOPGAL^0/0^ or **B**, BK5.ATF3^Tg/0^;TOPGAL^Tg/0^. Arrows, supra-basal tumor cells; asterisks, basal tumor cells.

Analysis of bacterial β-galactosidase expression by IHC was complicated by background cross-reactivity in ATF3-tumors induced in non-TOPGAL mice (Figure 3A). It can be seen that modest but fairly uniform staining was seen in the supra-basal tumor cells (arrows) but not in the basal cell layer of the tumors (asterisks). The antibody used for IHC is known to cross-react with mouse β-galactosidase, suggesting that the mouse protein may be present in the supra-basal layers of these tumors. In contrast, tumors arising in BK5.ATF3^Tg/0^;TOPGAL^Tg/0^ mice (Figure 3B) demonstrated uniform staining throughout both the basal and supra-basal layers of the tumor. We conclude that activation of the Wnt/β-catenin pathway occurs at least in the basal cell layers of these tumors, coincident with nuclear expression of ATF3 [21].

As a further test of Wnt/β-catenin pathway activation, we used qPCR and protein immunoblotting to monitor activity of known downstream transcriptional targets of this pathway. Wnt/β-catenin signaling is usually associated with increased cell proliferation, and correlated with this, *Ccnd1* and *Jun* have both been shown to be transcriptional targets of the Wnt/β-catenin pathway [38]–[40]. As shown in Figure 2B, the *Ccnd1* gene was up-regulated about four-fold in ATF3-induced tumors compared to normal mammary glands (p<0.001), and *Jun* expression was up about two-fold (p<0.001). Furthermore, at the protein level, robust up-regulation of both cyclin D1 and Jun proteins was seen in immunoblots (Figure 2C). Interestingly, immunoprecipitation of mammary tumor extracts with ATF3-specific antiserum, followed by immunoblotting demonstrated significant intracellular association between ATF3 and Jun proteins (Figure 2D). ATF3-Jun heterodimers have previously been shown to function as activators of gene expression, whereas ATF3 homodimers are generally thought to be repressors [3], [5], [6]. Jun must be phosphorylated at serine 73 in order to exhibit maximal activity as a transcriptional activator [41]. Using an antibody specific for phosphoserine-73, we noted high but variable levels of phosphorylated Jun in extracts from BK5.ATF3 transgenic mammary glands (Figure 2E, lanes 4–6) and from BK5.ATF3-induced mammary tumors (Figure 2E, lanes 7–9); much lower levels of activated Jun were seen in extracts from non-transgenic mammary glands (Figure 2E, lanes 1–3).

Transcriptional activation of the *Ccnd1* and *Jun* genes cannot be taken to be absolutely specific for the Wnt/β-catenin pathway, since both of these genes can be regulated by numerous other factors. However, several genes that are involved in the negative regulation of Wnt/β-catenin signaling, presumably as a homeostatic mechanism, are also known to be direct transcriptional targets of the pathway. *Axin2*, involved in negative regulation of the β-catenin protein through destabilization, is one such pathway-specific gene, known to be up-regulated by Wnt β-catenin signaling [42]. As shown in Figure 2B, mRNA for *Axin2* is expressed at four-fold higher levels in ATF3-induced tumors as compared to mammary glands (p<0.001). *Dkk4*, encoding a soluble protein that binds to Wnt receptors in the plasma membrane, but fails to activate downstream signaling, has also been shown to be a direct transcriptional target of Wnt/β-catenin pathway activation [43]. As shown in Figure 2B, *Dkk4* mRNA is barely detectable by qPCR in normal glands, but highly up-regulated in ATF3-induced tumors (p<0.01); the increase is at least 100-fold. In MMTV.*neu* mammary tumors in which the Wnt/β-catenin pathway is not involved, expression of *Axin2* was extremely low (relative expression 0.01 compared to BK5.ATF3 mammary glands, p<0.01, data not shown), and *Dkk4* was undetectable. These data strongly suggest that Wnt/β-catenin signaling is activated in BK5.ATF3 mammary tumors.

### Wnt ligands are over-expressed in BK5.ATF3 tumors

Canonical activation of the Wnt/β-catenin pathway begins with the binding of an activating Wnt protein to membrane receptors, followed by downstream cytoplasmic events that lead to stabilization of β-catenin, facilitating transport to the nucleus and transcriptional activation of target genes. Several Wnt genes that activate canonical Wnt signaling, including *Wnt2*, *Wnt5a*, *Wnt7b*, and *Wnt10b*, are known to be expressed in the mammary gland during ductal development [44], [45]. Thus, one possible mechanism for the activation of the Wnt β-catenin pathway by ATF3 would be direct activation of transcription of one or more *Wnt* genes. We analyzed the expression of 9 *Wnt* genes by qPCR in mammary tumors derived from parous BK5.ATF3 mice, and in normal, young adult mammary glands of non-transgenic and transgenic mice (Table 1). Five *Wnt* genes were significantly overexpressed in the tumors compared to transgenic mammary glands, including *Wnt3*, *Wnt3a*, *Wnt5a*, *Wnt7b* and *Wnt10b*. Notably, *Wnt3* was up-regulated over 70-fold in ATF3-induced tumors compared to transgenic mammary glands. Importantly, the expression of *Wnt3* was also 5-fold higher in transgenic glands than in non-transgenic glands. This may reflect a role for Wnt3 in the early stages of tumorigenesis in this model. None of the other Wnt genes assayed exhibited significantly higher expression in transgenic mammary glands compared to non-transgenic glands. Two genes, *Wnt1* and *Wnt 5b*, were significantly down-regulated in ATF3-induced mammary tumors.

**Table 1:** Expression of Wnt genes in mammary glands and tumors.

*Wnt* Gene	TG glands	Relative Expression: Tumors^a^	Relative Expression: WT glands
*Wnt3*	1.0±0.4	73±17***	0.2±0.1*
*Wnt3a*	1.0±0.3	33.6±12.3**	2.1±1.2
*Wnt5a*	1.0±0.6	7.2± 1.6***	1.3±0.5
*Wnt7b*	1.0±0.1	3.1±1.3**	2.0±0.9*
*Wnt10b*	1.0±0.5	2.4±0.4***	0.9±0.4
*Wnt4*	1.0±0.1	1.6±0.5	1.2±0.8
*Wnt9b*	1.0±1.0	1.3±0.8	0.7±0.4
*Wnt5b*	1.0±0.6	0.2±0.1*	1.5±0.4
*Wnt1*	1.0±0.8	0.03±0.01*	0.8±0.6

a All data are expressed relative to the level of expression found in transgenic mammary glands. Statistical significance, determined by Student's t-test, is indicated as follows:

*, p<0.05;

**, p<0.01;

***, p<0.001.

### Snai1 and Snai2 are highly expressed in BK5.ATF3 tumors

**Figure 4:**
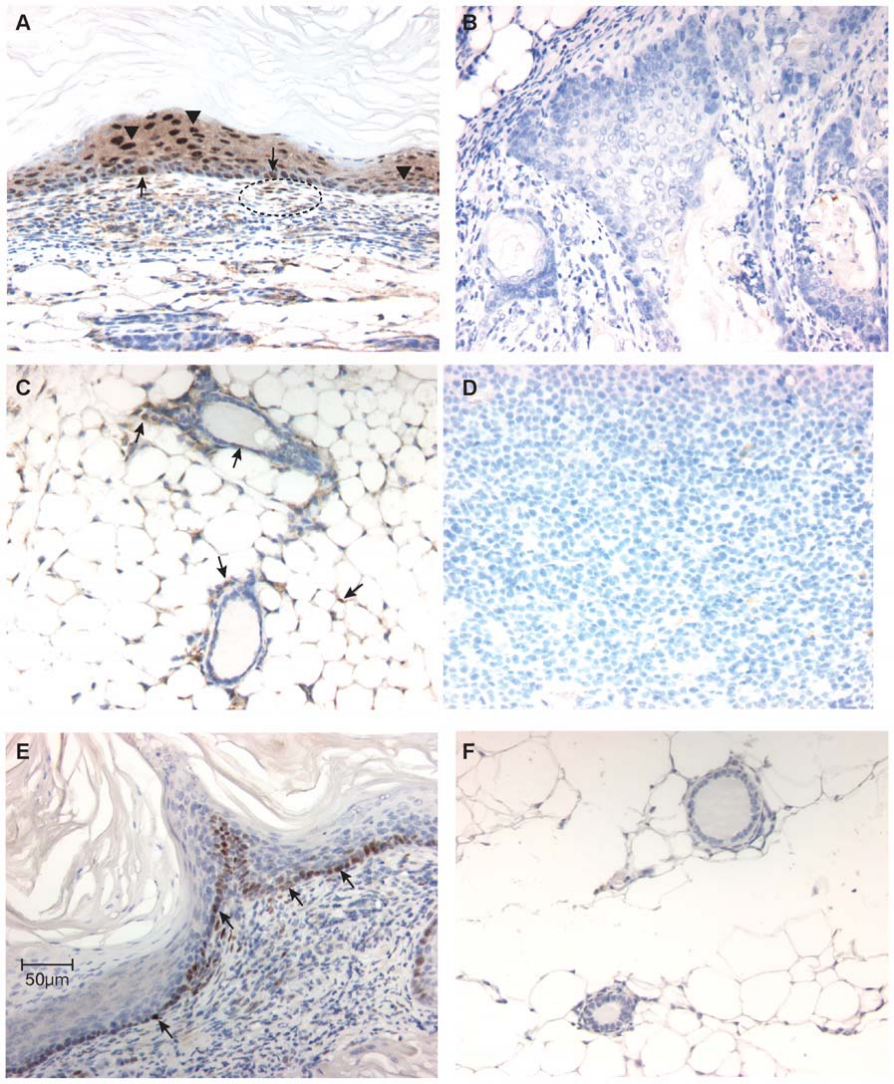
*Snai1* and *Snai2* are expressed in ATF3-induced tumors. Immunohistochemical analysis of Snail (coded by the *Snai1* gene) expression in: **A,B**, BK5.ATF3 mammary tumors; **C**, normal mammary gland of a non-transgenic female; **D**, a mammary tumor from a MMTV.neu transgenic female. In panel **B**, a blocking peptide specific for Snail was incubated with the anti-Snail antibody as a negative control. Arrows, labeled basal cell nuclei; arrowheads, labeled supra-basal cell nuclei. Immunohistochemical analysis of Slug (coded by the *Snai2* gene) in **E**, a BK5.ATF3 mammary tumor and **F**, an apparently normal mammary gland from a tumor-bearing BK5.ATF3 female. Scale bar in **E** = 50 µm, applies to all panels.

The *SNAI1* and *SNAI2* genes have been implicated in tumor progression and metastasis [46]–[49], and both were recently shown to be direct transcriptional target of ATF3 in human mammary cells [19]. The transcriptional repression activity of Snail and Slug proteins [49] is expected to positively impact the canonical Wnt/β-catenin pathway. However, the possible involvement of these genes in the early phases of mammary tumorigenesis has not been demonstrated. IHC analysis indicated robust expression of Snail protein (the product of *Snai1*) in ATF3-induced mammary tumors (Figure 4A). Nuclei of both basal and supra-basal epithelial tumor cells (arrows and arrowheads, respectively) exhibited Snail expression. In addition, scattered nuclei throughout the stroma were strongly positive for Snail expression (dotted line). Nuclear staining for Snail was not seen in control sections in which a blocking peptide for Snail1 was included in the incubation (Figure 4B). Scattered Snail-positive nuclei were observed in normal mammary glands, in both the epithelial and stromal compartments (Figure 4C). Essentially no expression of Snail was seen in mammary tumors that developed in MMTV.neu transgenic animals (Figure 4D). Further quantitative analysis of five independent tumors indicated that 80–90% of tumor cell nuclei were positive for Snail expression. Consistent with this, qPCR analysis indicated about a four-fold up-regulation of *Snai1* mRNA in these tumors compared to normal mammary glands in BK5.ATF3 animals (Figure 2A, p<0.001). Slug (the protein product of *Snai2*) was also expressed in the nuclei of tumor cells, but in this case was seen primarily in the basal cell layers of the tumors (Figure 4E). Quantitatively, only about 28% of the tumor cells exhibited Slug expression; no staining was seen in normal mammary glands (Figure 4F). Scattered stromal cells also exhibited Slug expression (Figure 4E, dotted line). qPCR analysis of *Snai2* expression failed to show a significant difference between transgenic mammary glands and mammary tumors (data not shown). This failure to detect increased levels of *Snai2* mRNA may be due to the restricted pattern of expression of Slug in basal tumor cells only, as visualized by IHC.

### Knock-down of ATF3 decreases expression of Wnt pathway genes

**Figure 5:**
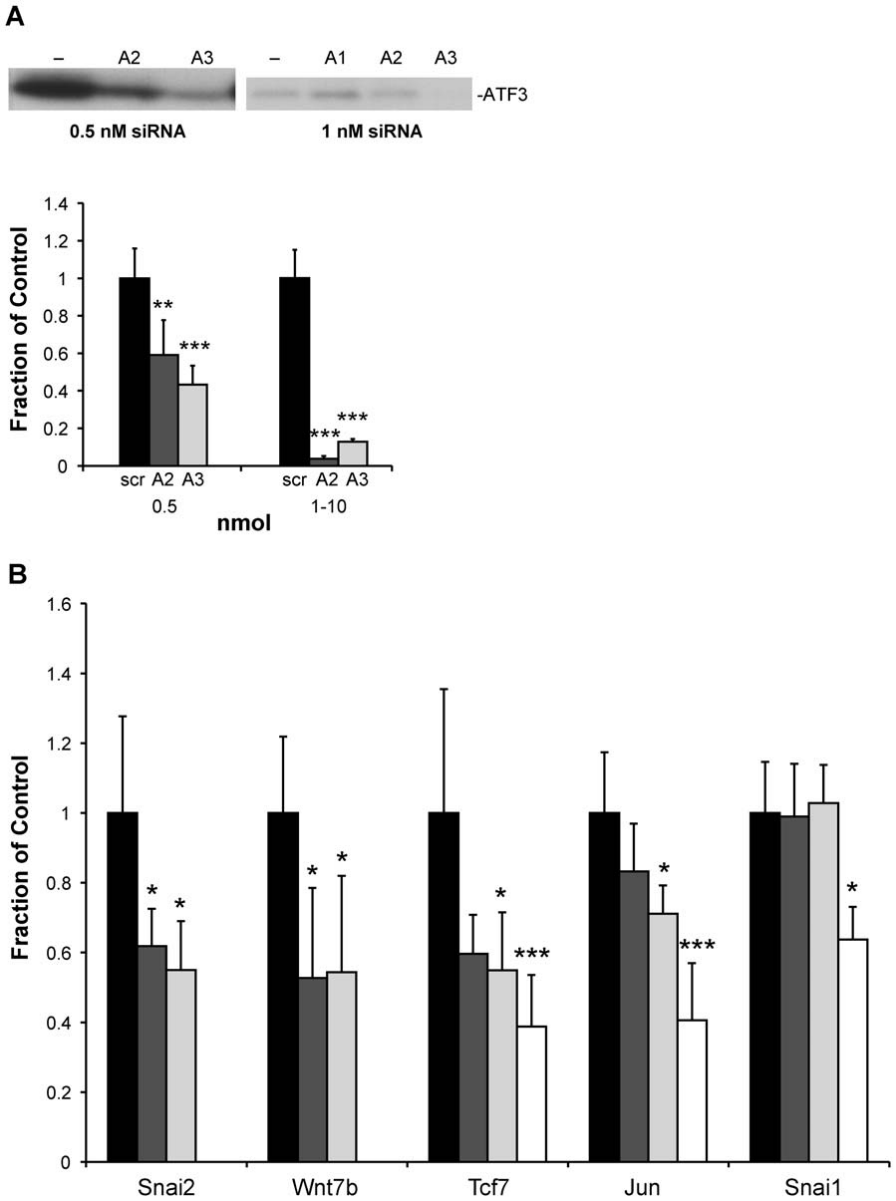
Decreases in gene expression following ATF3 knock-down in EMT6 cells. EMT6 cells were transfected with silencing RNAs A1, A2, or A3 as described in Materials and Methods, or with a scrambled version of A1, at the indicated concentrations. **A**, 48 h later extracts were analyzed for ATF3 protein expression by immunoblotting (upper panels) or for mRNA expression by qPCR (lower panel). Gene expression levels are expressed relative to expression in cells treated with the scrambled siRNA. The statistical significance of differences between mean expression levels, calculated relative to the scrambled siRNA control, is indicated over the bars of the histograms: *, p<0.05: **, p<0.01: ***, p<0.001. **B**, qPCR was used to analyze the same extracts for *Snai2*, *Wnt7b*, *Tcf7*, *Jun*, and *Snai1* as indicated. Black bars, scrambled siRNA; dark gray bars, 0.5 nmol A2siRNA; light gray bars, 0.5 nmol A3siRNA; white bars, 1–10 nmol A2siRNA.

Many of the changes in gene expression seen in ATF3-induced mammary tumors may not be due to direct effects of ATF3 on the transcription of the affected gene, but to indirect effects related to changes in signaling pathways. We would like to know which, if any, of the affected genes are direct targets of ATF3. As an initial approach, we utilized *in vitro* transfection with siRNA to knock-down Atf3 expression, and then measured the effects on the expression of potential target genes. For these experiments, we utilized a mouse mammary cancer cell line, EMT6, in which we found robust expression of the endogenous *Atf3* gene in preliminary experiments. We identified three sequences from the *Atf3* mRNA that could potentially serve as targets for gene silencing. As shown in Figure 5A, expression of ATF3 protein could be knocked down in EMT6 cells by treatment with two different siRNA sequences, A2 and A3, but not with a third siRNA (A1). When assayed at the mRNA level by qPCR (Figure 5A), the lowest dose of siRNAs A2 or A3 (0.5 nM) decreased the expression level of the endogenous Atf3 mRNA by about 50% compared to treatment with a scrambled version of siRNA A1, while higher doses (1, 3, or 10 nM) gave ∼90% knockdown.

In untreated EMT6 cells, we assayed expression of 15 endogenous mouse genes related to Wnt/β-catenin signaling and/or previously identified as up-regulated in ATF3-induced tumors. Expression of several genes of interest (*Wnt3*, *Wnt3a* and *Dkk4*) was undetectable in these cells. However, six genes identified as up-regulated in BK5.ATF3-induced tumors or transgenic mammary glands were easily detected by qPCR (*Jun*, *Snai1*, *Snai2*, *Wnt7b*, *Wnt10b* and *Tcf7*), and were chosen for further studies. When *Atf3* mRNA expression was knocked down by ∼50% using either siRNA A2 or A3, statistically significant 40–45% decreases in the expression of both *Snai2* and *Wnt7b* were seen (Figure 5B); in all cases, decreases were calculated relative to treatment with a scrambled siRNA that produced no knock-down of *Atf3* expression. Expression of *Tcf7* was similarly decreased by A3 under these conditions. On the other hand, expression of *Wnt10b* was increased significantly by *Atf3* knock-down with both A2 and A3 (data not shown). With higher doses of siRNA A2, where *Atf3* expression was knocked down >90%, about a 60% decrease in *Jun* expression and a 35% decrease in *Snai1* expression were also seen (Figure 5B). *SNAI1* and *SNAI2*, the human orthologues of *Snai1* and *Snai2*, have previously been shown to be direct transcriptional targets of ATF3 [19], but transcriptional regulation of the remaining genes by ATF3 has not been described.

**Figure 6:**
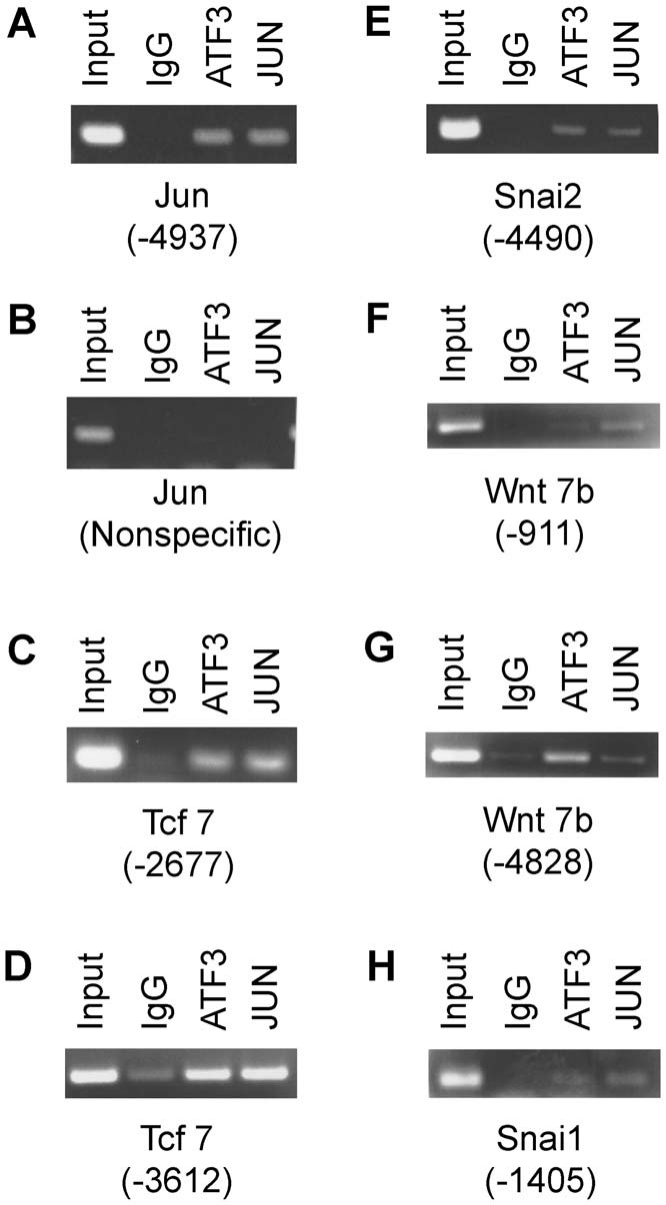
ChIP analysis of ATF3 and JUN binding to selected AP-1 sites. Nuclei from untreated EMT6 cells were prepared for chromatin immunoprecipitation as described in Materials and Methods, and precipitated with antibodies specific for ATF3 or JUN, or with a non-specific IgG. DNA was purified from the precipitates and used as template in PCR reactions designed to amplify fragments containing AP-1 sites at the indicated distances upstream of the murine genes for: **A**, *Jun*; **C,D**, *Tcf7*; **E**, *Snai2*; **F,G**, *Wnt7b*; **H**, *Snai1*. In panel **B**, the PCR targeted a non-specific site over 6000 bp upstream from the *Jun* transcriptional start site that did not contain an AP-1 site. In each panel, a small fraction of the starting DNA (2%) was used as template for the reaction in the first lane (Input), and the remaining lanes used the indicated immunoprecipitated DNA preparations (IgG, ATF3 and JUN).

As noted above for ATF3-expressing mammary glands, it is possible that the high levels of expression of Atf3 and Jun in untreated EMT6 cells promote formation of Atf3:Jun heterodimers. Such heterodimers exhibit AP-1-like activity [3], activating transcription through DNA binding sites with the consensus sequence (A/G)TGA(G/C)T(C/A)A. For several genes that are highly expressed in EMT6 cells (*Jun*, *Tcf7*, *Snai2*, *Wnt7b* and *Snai1*), we noted the presence in the DNA sequence upstream of the transcriptional start site of one (*Jun*, *Snai2*, *Snai1*) or two (*Tcf7*, *Wnt7b*) close matches (Table 2) to a particular subclass of AP-1 sites (GTGA(G/C)TCA). As a preliminary test of whether binding of ATF3 and/or JUN to these AP-1 sites might be involved in their expression in EMT6 cells, we performed ChIP experiments with antibodies against either ATF3 or JUN. For each of these genes, PCR signals for the expected fragment(s) containing the putative AP-1 binding site were obtained with chromatin precipitated with either an ATF3-specific or a JUN-specific antibody (Figure 6). The strongest PCR products in the ChIP experiment were obtained for the putative AP-1 sites upstream of *Jun* (panel A), *Tcf7* (panels C,D) and *Snai2* (panel E), for which the target sequence exactly matched the GTGA(G/C)TCA motif (Table 2). No PCR product was visible with either antibody in an assay directed at a non-specific site upstream of *Jun* (panel B) that had no match to this motif. The AP-1 sites upstream of *Wnt7b* and *Snai1* contained one to three mismatches to the consensus motif (Table 2), and the positive PCR signals obtained in the corresponding ChIP samples (Figure 6, f–h) were somewhat weaker. These results are consistent with the suggestion that *Snai2*, *Snai1*, *Tcf7*, *Jun* and *Wnt7b* may be direct transcriptional targets of ATF3 and JUN, possibly binding as a heterodimer to upstream enhancer elements.

**Table 2:** Putative AP-1 sites.

Gene	Position	Sequence
*Jun*	−4937	GTGACTCA
*Tcf7*	−2677	GTGACTCA
*Tcf7*	−3612	GTGACTCA
*Snai2*	−4920	GTGACTCA
*Wnt7b*	−911	GTGAGaCA
*Wnt7b*	−4828	GTGAtTaA
*Snai1*	−1405	cTtAGTaA

## Discussion

In the present communication, we have provided several lines of evidence that the canonical Wnt/β-catenin pathway is activated in BK5.ATF3 mammary tumors. Characteristic overexpression of the β-catenin protein and its appearance in nuclei of tumor cells is seen. Downstream transcriptional targets, including *Ccnd1*, *Jun*, *Axin2* and *Dkk4*, are up-regulated, and a bacterial reporter gene that is driven by Wnt/β-catenin signaling is activated in the tumors. In addition, the genes for several positive-acting ligands for the pathway, including *Wnt3*, *Wnt3a*, *Wnt7b* and *Wnt10b*, are significantly over-expressed in tumors. Given the known importance of Wnt/β-catenin signaling in colorectal cancer [29], [30], and accumulating evidence suggesting that this pathway is often dysregulated in human breast cancer [31]–[34], [50], [51], it seems likely that Wnt/β-catenin signaling is important in the genesis of mammary tumors in this mouse model. Experiments to test this suggestion by blocking Wnt/β-catenin signaling in the transgenic mice are in progress. This is consistent with results in several other transgenic models [22]–[28] in which Wnt/β-catenin pathway activation results in mammary tumors or preneoplastic lesions. Several of these exhibit the tendency for squamous differentiation that is extremely prominent within the ATF3-induced tumors.

There have been no previous reports linking ATF3 expression to Wnt/β-catenin pathway activation. However, our finding that the genes for several positive-acting Wnt ligands (*Wnt3*, *Wnt3a*, *Wnt7b* and *Wnt10b*) are up-regulated in the tumors suggests the obvious possibility that ATF3 may be acting as a direct transcriptional regulator of one or more of these ligands. Consistent with this, we find that knockdown of ATF3 expression *in vitro* reduces *Wnt7b* expression and ChIP analysis in EMT6 cells suggests that both AFT3 & JUN proteins bind to regions upstream of the Wnt7b promoter that contain putative AP-1 consensus motifs. Other positive ligands, particularly Wnt3 and Wnt3a which are highly up-regulated in ATF3-induced tumors, may also be important in tumorigenesis, but are expressed at very low levels in EMT6 cells and therefore could not be tested in the knock-down experiments. This model is attractive in that it provides an explanation for the finding that both basal and supra-basal tumor cells exhibit nuclear β-catenin expression, while only basal cells over-express ATF3. Because Wnt ligands are secreted and act extra-cellularly [35], both autocrine and paracrine stimulation of the pathway in the basal and supra-basal cells, respectively, might reasonably be expected to occur, leading to the pattern of nuclear β-catenin expression seen.

Non-parous, BK5.ATF3 females do not develop mammary tumors [21], and their mammary glands do not express high levels of β-catenin mRNA (Figure 2B), do not exhibit nuclear localization of β-catenin (Figure 1A), and are unable to activate the TOPGAL reporter gene (Figure 2A). Thus, although ATF3 is clearly expressed throughout post-natal development in the basal cell compartment of mammary glands in BK5.ATF3 transgenic females, this overexpression by itself in not sufficient to produce tumors, nor to fully activate the Wnt/β-catenin pathway. The effects of ATF3 expression in other systems are strongly context-dependent [3], [19], and can include both apoptosis [13] and growth stimulation [14]–[17], oncogenesis [18]–[21] and tumor suppression [12]. The requirement for parity to induce mammary tumorigenesis in the current model suggests that during pregnancy, lactation and/or involution contextual changes occur that allow full Wnt/β-catenin pathway activation to occur. Preliminary histopathological analyses (data not shown) have revealed that at mid-lactation, lobulo-alveolar differentiation is incomplete in transgenic glands, and further studies of this process and of involution at both the histological and molecular levels are in progress.

The *SNAI2* and *SNAI1* genes have recently been identified as direct transcriptional targets of ATF3 in human mammary cells [19], and the murine homologs, *Snai2* and *Snai1* are up-regulated in ATF3-induced mammary tumors (Figure 4). Multiple, bi-directional interactions between Wnt/β-catenin pathway activation and Snail have been identified previously. Snail interacts directly with nuclear β-catenin [52] and indirectly through repression of E-cadherin [49] to increase the transactivation capacity of β-catenin. Wnt pathway activation, on the other hand, up-regulates Snail activity by stabilizing nuclear Snail protein in an Axin2/GSK3β-mediated process [53]. Thus, a direct transcriptional activation of Snail by ATF3 may also be important in activating and/or maintaining Wnt/β-catenin signaling in these tumors through a positive feedback loop.

Global gene expression analyses of human breast tumors have identified an expression pattern consistent with up-regulation of the canonical Wnt/β-catenin pathway that associates with the basal-like tumor subclass [54], [55]. Since there is significant overlap between the basal-like subclass and clinically defined triple negative breast cancer [56], [57], this implies that Wnt/β-catenin signaling is important in triple negative breast cancer, representing those breast cancers that have the worst prognosis and no effective treatment regimen. Importantly, Rosen's laboratory has recently shown that activation of Wnt/β-catenin signaling is a hallmark of tumor-initiating cells in a mouse model [58]. Thus we have reason to believe that further analysis of the BK5.ATF3 model, in which mammary tumors are clearly basal-like and exhibit activated Wnt/β-catenin signaling, may provide a better understanding of processes that are extremely important in human breast tumorigenesis.

## Materials and Methods

Detailed methods for IHC, protein immunoblotting, qPCR, RNA silencing and ChIP analysis are given in Supplementary Materials and Methods (Text S1).

### Animals

Mice were maintained in a light and temperature controlled room in an AAALAC-accredited facility, and given water and lab chow *ad libitum*. All experimental procedures were approved by the Institutional Animal Care and Use Committee of The University of Texas MD Anderson Cancer Center under protocol #05-01-03934. The derivation of the BK5.ATF3 transgenic mice has been described [20]. TOPGAL mice and MMTV.neu mice were obtained from Jackson Laboratory (Bar Harbor, ME). Genotyping was done with appropriate PCR assays, using DNA purified from tail snips.

A tumor experiment with female mice that were hemizygous for both the BK5.ATF3 and TOPGAL transgenes was performed by first allowing the mice to mate and raise pups twice before the age of 6 months. Parous females were palpated twice weekly and were euthanized when palpable tumors reached 1.5 cm in diameter or when animals became moribund. The experiment was terminated at 16 months.

### Cell culture

The EMT6 line of murine mammary cancer cells (ATCC: CRL-2755TM) was maintained in Waymouth's MB 752/1 medium with 2 nM L-glutamine and 15% fetal bovine serum at 5% CO2, and 37°C.

### RNA purification and qPCR

Total RNA was isolated using TRIzol reagent (Invitrogen, Grand Island, NY) according to the manufacturer's protocol, and reverse transcribed using the High Capacity cDNA Archive kit (Applied Biosystems, Foster City, CA). Real-time PCR was performed with an ABI Prism 7900HT Sequence Detection System (Applied Biosystems). Student's t-test (two-tailed, assuming unequal variance) was used to determine statistical significance (p-values) for differences in the relative expression values between tissues for each gene analyzed. p-values less than 0.05 were considered significant.

## Supporting Information

Text S1 **Supplementary Materials and Methods**
(DOC)

Figure S1 **Mammary tumorigenesis in parous BK5.ATF3; TOPGAL mice.** Singly transgenic animals from the BK5.ATF3 line and the TOPGAL line were mated to produce double heterozygotes (ATF3^+/−^, TOPGAL^+/−^). The doubly heterozygous females and their singly transgenic (ATF3^−/−^, TOPGAL^+/−^) littermates were allowed to mate and raise litters twice, and then monitored for mammary tumor formation until 16 months of age. Tumor-bearing animals were sacrificed when a tumor reached 1.5 cm in its longest dimension. **A.** Survival curves are shown for several different genotypes. Data for TOPGAL non-TG/ATF3 TG mice is from reference (Wang *et al*., 2008). **B.** Tumors arising in (ATF3^+/−^, TOPGAL^+/−^) females were harvested and analyzed by histopathology. Scale bar in panel B = 50 µm.
(TIF)

## References

[1] Allan AL, Albanese C, Pestell RG, LaMarre J (2001). Activating transcription factor 3 induces DNA synthesis and expression of cyclin D1 in hepatocytes.. J Biol Chem.

[2] Hai T, Wolfgang CD, Marsee DK, Allen AE, Sivaprasad U (1999). ATF3 and stress responses.. Gene Expr.

[3] Hsu JC, Bravo R, Taub R (1992). Interactions among LRF-1, JunB, c-Jun and c-Fos define a regulatory program in the G1 phase of liver regeneration.. Mol Biol Cell.

[4] Wolfgang CD, Chen BP, Martindale JL, Holbrook NJ, Hai T (1997). gadd153/Chop10, a potential target gene of the transcriptional repressor ATF3.. Mol Cell Biol.

[5] Wolfgang CD, Liang G, Okamoto Y, Allen AE, Hai T (2000). Transcriptional autorepression of the stress-inducible gene ATF3.. J Biol Chem.

[6] Chen BP, Liang G, Whelan J, Hai T (1994). ATF3 and ATF3 delta Zip. Transcriptional repression versus activation by alternatively spliced isoforms.. J Biol Chem.

[7] Fan F, Jin S, Amundson SA, Tong T, Fan W (2002). ATF3 induction following DNA damage is regulated by distinct signaling pathways and over-expression of ATF3 protein suppresses cells growth.. Oncogene.

[8] Hartman MG, Lu D, Kim ML, Kociba GJ, Shukri T (2004). Role for activating transcription factor 3 in stress-induced beta-cell apoptosis.. Mol Cell Biol.

[9] Kool J, Hamdi M, Cornelissen-Steijger P, van der Eb AJ, Terleth C (2003). Induction of ATF3 by ionizing radiation is mediated via a signaling pathway that includes ATM, Nibrin1, stress-induced MAPkinases and ATF-2.. Oncogene.

[10] Hamdi M, Popeijus HE, Carlotti F, Janssen JM, van der Burgt C (2008). ATF3 and Fra1 have opposite functions in JNK- and ERK-dependent DNA damage responses.. DNA Repair (Amst).

[11] Yan C, Wang H, Boyd DD (2002). ATF3 Represses 72-kDa Type IV Collagenase (MMP-2) Expression by Antagonizing p53-dependent trans-Activation of the Collagenase Promoter.. J Biol Chem.

[12] Lu D, Wolfgang CD, Hai T (2006). Activating transcription factor 3, a stress-inducible gene, suppresses Ras-stimulated tumorigenesis.. J Biol Chem.

[13] Turchi L, Aberdam E, Mazure N, Pouyssegur J, Deckert M (2008). Hif-2alpha mediates UV-induced apoptosis through a novel ATF3-dependent death pathway.. Cell Death Differ.

[14] Janz M, Hummel M, Truss M, Wollert-Wulf B, Mathas S (2006). Classical Hodgkin lymphoma is characterized by high constitutive expression of activating transcription factor 3 (ATF3), which promotes viability of Hodgkin/Reed-Sternberg cells.. Blood.

[15] Kawauchi J, Zhang C, Nobori K, Hashimoto Y, Adachi MT (2002). Transcriptional repressor activating transcription factor 3 protects human umbilical vein endothelial cells from tumor necrosis factor-alpha-induced apoptosis through down-regulation of p53 transcription.. J Biol Chem.

[16] Nakagomi S, Suzuki Y, Namikawa K, Kiryu-Seo S, Kiyama H (2003). Expression of the activating transcription factor 3 prevents c-Jun N-terminal kinase-induced neuronal death by promoting heat shock protein 27 expression and Akt activation.. J Neurosci.

[17] Nobori K, Ito H, Tamamori-Adachi M, Adachi S, Ono Y (2002). ATF3 inhibits doxorubicin-induced apoptosis in cardiac myocytes: a novel cardioprotective role of ATF3.. J Mol Cell Cardiol.

[18] Perez S, Vial E, van Dam H, Castellazzi M (2001). Transcription factor ATF3 partially transforms chick embryo fibroblasts by promoting growth factor-independent proliferation.. Oncogene.

[19] Yin X, Dewille JW, Hai T (2008). A potential dichotomous role of ATF3, an adaptive-response gene, in cancer development.. Oncogene.

[20] Wang A, Arantes S, Conti C, McArthur M, Aldaz CM (2007). Epidermal hyperplasia and oral carcinoma in mice overexpressing the transcription factor ATF3 in basal epithelial cells.. Mol Carcinog.

[21] Wang A, Arantes S, Yan L, Kiguchi K, McArthur MJ (2008). The transcription factor ATF3 acts as an oncogene in mouse mammary tumorigenesis.. BMC Cancer.

[22] Farago M, Dominguez I, Landesman-Bollag E, Xu X, Rosner A (2005). Kinase-inactive glycogen synthase kinase 3beta promotes Wnt signaling and mammary tumorigenesis.. Cancer Res.

[23] Imbert A, Eelkema R, Jordan S, Feiner H, Cowin P (2001). Delta N89 beta-catenin induces precocious development, differentiation, and neoplasia in mammary gland.. J Cell Biol.

[24] Li Q, Dashwood WM, Zhong X, Al-Fageeh M, Dashwood RH (2004). Cloning of the rat beta-catenin gene (Ctnnb1) promoter and its functional analysis compared with the Catnb and CTNNB1 promoters.. Genomics.

[25] Miyoshi K, Shillingford JM, LeProvost F, Gounari F, Bronson R (2002). Activation of β-catenin signaling in differentiated mammary secretory cells induces transdifferentiation into epidermis and squamous metaplasias.. Proc Natl Acad Sci U S A.

[26] Miyoshi K, Rosner A, Nozawa M, Byrd C, Morgan F (2002). Activation of different Wnt/b-catenin signaling components in mammary epithelium induces transdifferentiation and the formation of pilar tumors.. Oncogene.

[27] Rosner A, Miyoshi K, Landesman-Bollag E, Xu X, Seldin DC (2002). Pathway pathology: histological differences between ErbB/Ras and Wnt pathway transgenic mammary tumors.. Am J Pathol.

[28] Teuliere J, Faraldo MM, Deugnier MA, Shtutman M, Ben-Ze'ev A (2005). Targeted activation of beta-catenin signaling in basal mammary epithelial cells affects mammary development and leads to hyperplasia.. Development.

[29] Kinzler KW, Vogelstein B (1996). Lessons from Hereditary Colorectal Cancer.. Cell.

[30] Bienz M, Clevers H (2000). Linking colorectal cancer to Wnt signaling.. Cell.

[31] Jin Z, Tamura G, Tsuchiya T, Sakata K, Kashiwaba M (2001). Adenomatous polyposis coli (APC) gene promoter hypermethylation in primary breast cancers.. Br J Cancer.

[32] Virmani AK, Rathi A, Sathyanarayana UG, Padar A, Huang CX (2001). Aberrant methylation of the adenomatous polyposis coli (APC) gene promoter 1A in breast and lung carcinomas.. Clin Cancer Res.

[33] Suzuki H, Toyota M, Carraway H, Gabrielson E, Ohmura T (2008). Frequent epigenetic inactivation of Wnt antagonist genes in breast cancer.. Br J Cancer.

[34] Veeck J, Noetzel E, Bektas N, Jost E, Hartmann A (2008). Promoter hypermethylation of the SFRP2 gene is a high-frequent alteration and tumor-specific epigenetic marker in human breast cancer.. Mol Cancer.

[35] Logan CY, Nusse R (2004). The Wnt signaling pathway in development and disease.. Annu Rev Cell Dev Biol.

[36] Muller W, Sinn E, Pattengale P, Wallace R, Leder P (1988). Single-step induction of mammary adenocarcinoma in transgenic mice bearing the activated c-neu oncogene.. Cell.

[37] DasGupta R, Fuchs E (1999). Multiple roles for activated LEF/TCF transcription complexes during hair follicle development and differentiation.. Development.

[38] Shtutman M, Zhurinsky J, Simcha I, Albanese C, D'Amico M (1999). The cyclin D1 gene is a target of the beta-catenin/LEF-1 pathway.. Proc Natl Acad Sci U S A.

[39] Tetsu O, McCormick F (1999). Beta-catenin regulates expression of cyclin D1 in colon carcinoma cells.. Nature.

[40] Mann B, Gelos M, Siedow A, Hanski ML, Gratchev A (1999). Target genes of beta-catenin-T cell-factor/lymphoid-enhancer-factor signaling in human colorectal carcinomas.. Proc Natl Acad Sci U S A.

[41] Smeal T, Hibi M, Karin M (1994). Altering the specificity of signal transduction cascades: positive regulation of c-Jun transcriptional activity by protein kinase A.. EMBO J.

[42] Jho EH, Zhang T, Domon C, Joo CK, Freund JN (2002). Wnt/beta-catenin/Tcf signaling induces the transcription of Axin2, a negative regulator of the signaling pathway.. Mol Cell Biol.

[43] Bazzi H, Fantauzzo KA, Richardson GD, Jahoda CA, Christiano AM (2007). The Wnt inhibitor, Dickkopf 4, is induced by canonical Wnt signaling during ectodermal appendage morphogenesis.. Dev Biol.

[44] Weber-Hall SJ, Phippard DJ, Neimeyer CC, Dale TC (1994). Developmental and hormonal regulation of Wnt gene expression in the mouse mammary gland.. Differentiation.

[45] Gavin BJ, McMahon AP (1992). Differential regulation of the Wnt gene family during pregnancy and lactation suggests a role in postnatal development of the mammary gland.. Mol Cell Biol.

[46] Storci G, Sansone P, Trere D, Tavolari S, Taffurelli M (2008). The basal-like breast carcinoma phenotype is regulated by SLUG gene expression.. J Pathol.

[47] Come C, Magnino F, Bibeau F, De Santa Barbara P, Becker KF (2006). Snail and slug play distinct roles during breast carcinoma progression.. Clin Cancer Res.

[48] Moody SE, Perez D, Pan TC, Sarkisian CJ, Portocarrero CP (2005). The transcriptional repressor Snail promotes mammary tumor recurrence.. Cancer Cell.

[49] Nieto MA (2002). The snail superfamily of zinc-finger transcription factors.. Nat Rev Mol Cell Biol.

[50] Hayes MJ, Thomas D, Emmons A, Giordano TJ, Kleer CG (2008). Genetic changes of Wnt pathway genes are common events in metaplastic carcinomas of the breast.. Clin Cancer Res.

[51] Veeck J, Bektas N, Hartmann A, Kristiansen G, Heindrichs U (2008). Wnt signalling in human breast cancer: expression of the putative Wnt inhibitor Dickkopf-3 (DKK3) is frequently suppressed by promoter hypermethylation in mammary tumours.. Breast Cancer Res.

[52] Stemmer V, de Craene B, Berx G, Behrens J (2008). Snail promotes Wnt target gene expression and interacts with beta-catenin.. Oncogene.

[53] Yook JI, Li XY, Ota I, Hu C, Kim HS (2006). A Wnt-Axin2-GSK3beta cascade regulates Snail1 activity in breast cancer cells.. Nat Cell Biol.

[54] Smid M, Wang Y, Zhang Y, Sieuwerts AM, Yu J (2008). Subtypes of breast cancer show preferential site of relapse.. Cancer Res.

[55] DiMeo TA, Anderson K, Phadke P, Fan C, Perou CM (2009). A novel lung metastasis signature links Wnt signaling with cancer cell self-renewal and epithelial-mesenchymal transition in basal-like breast cancer.. Cancer Res.

[56] Bertolo C, Guerrero D, Vicente F, Cordoba A, Esteller M (2008). Differences and molecular immunohistochemical parameters in the subtypes of infiltrating ductal breast cancer.. Am J Clin Pathol.

[57] Kreike B, van Kouwenhove M, Horlings H, Weigelt B, Peterse H (2007). Gene expression profiling and histopathological characterization of triple-negative/basal-like breast carcinomas.. Breast Cancer Res.

[58] Zhang M, Atkinson RL, Rosen JM (2010). Selective targeting of radiation-resistant tumor-initiating cells.. Proc Natl Acad Sci U S A.

