# Supplementary material for: Activation of the Canonical Wnt/β-Catenin Pathway in ATF3-Induced Mammary Tumors
Source: PLoS One. 2011 Jan 31;6(1):e16515. doi: 10.1371/journal.pone.0016515 (PMC3031586; doi:10.1371/journal.pone.0016515)
Supplement: Text S1 — Supplementary Materials and Methods (DOC) [file pone.0016515.s001.doc]

**Supplementary Materials and Methods**

RNA purification and qPCR

Total RNA was isolated from cell cultures, mouse mammary glands and mammary tumors (derived either from BK5.ATF3 or MMTV.neu transgenic mice) using TRIzol reagent (Invitrogen) according to the manufacturer’s protocol. RNA integrity was checked by RNA 6000 Nano Chip (Agilent Bioanalyzer, Santa Clara, CA). Mammary glands from BK5.ATF3 mice were in all cases obtained from virgin females between 6 and 8 months of age, to ensure that the transgenic glands did not contain regions with preneoplastic or early neoplastic lesions. Nontransgenic glands were age-matched.

For tissue and tumor RNA, two microgram of total RNA from each sample tissue was reverse-transcribed into cDNA by using the High Capacity cDNA Archive kit (Applied Biosystems). Real-time PCR was performed using cDNA with TaqMan Universal PCR Master mix on an ABI Prism 7900HT Sequence Detection System (Applied Biosystems). To measure expression of the bacterial β-galactosidase reporter gene, driven by Wnt/β-catenin pathway activation, a Customized TaqMan® Gene Expression Assay was used. Primers and probe for this assay were as follows: forward primer: GGCGGAAAACCTCAGTGTGA; reverse primer: GCTTATTACCCAGCTCGATGCAA; FAM-labeled probe: CCATCCCGCATCTGAC. The ID numbers for all other individual TaqMan® Gene Expression Assays used were as follows: Atf3(Mm00476032_m1), Ctnnb1(Mm00483039_m1), Ccnd1(Mm00432359_m1), Jun(Mm00495062_s1), Axin2(Mm00443610_m1), Snai1(Mm00441533_m1), Snai2(Mm00441531_m1) and (Mm00441531_m1), Dkk4(Mm00461141_m1), Lef1(Mm00550265_m1), Tcf7(Mm00493445_m1), Tcf7l2(Mm00501505_m1), Wnt1(Mm01300555_g1), Wnt3(Mm00437336_m1), Wnt3a(Mm00437337_m1) and (Mm03053669_s1), Wnt4(Mm01194003_m1), Wnt5a(Mm00437347_m1), Wnt5b(Mm00437350_m1), Wnt7b(Mm00437357_m1), Wnt9b(Mm00457102_m1), Wnt10b(Mm00442104_m1), Tcf7l1(Mm01188714_m1). Human Eukaryotic 18S rRNA (part # 4352930E) was used as endogenous control for relative gene expression quantification. The relative standard curve method was used to calculate the fold-difference in target gene expression between mammary tumor and mammary gland.

For experiments with EMT6 cells, 400 ng total RNA, extracted using TRIzol, was reverse transcribed into cDNA. TaqMan® Gene Expression Assay for mouse GAPDH(Applied Biosystems, part # 4352339E) was used as the endogenous control for relative gene expression quantification, and the comparative CT Method (ΔΔCt) was used to calculate the relative fold- difference.

RNA silencing

We used three different ATF3 siRNAs: ATF3 silencer® select pre-designed siRNA (ID: s62686, s62687 and s62688) together with silencer® select negative control #1 siRNA (cat# 4390843). All siRNA were from Applied Biosystems (Foster City, CA).

EMT6 cells were plated at 20,000 cells/well in 24-well plates, 24 h before transfection. 4 μl siPORT Amine (Applied Biosystems) was diluted into 25 μl OPTI-MEM I (Invitrogen, Grand Island, NY) for each well. Each siRNA was also diluted in OPTI-MEM I medium for a final concentration of 0.5 nM, 1nM, 3nM or 10 nM in transfection. Diluted RNA and transfection agent were mixed and added to the cells following the manufacturer’s suggested protocol. RNA was extracted 48 h after transfection, and assayed for target-gene expression as described below.

IHC, protein immunoblots

Immunohistochemistry assays were performed by the Tissue Processing Facility Core of the Center for Research on Environmental Disease as described previously. The sources of the primary antibodies used were as follows: β-galactosidase (Chemicon [Tomecula, CA]:AB1211); β-catenin (BD/Transduction Laboratories [Franklin Lakes, NJ]:610153); Snail (Abcam [Cambridge, MA]:17732); Slug (Cell Signaling [Danvers, MA]: 9585).

Nuclear extracts of the fourth and fifth mammary glands and tumors from BK5-ATF3 transgenic mice were prepared by using NE-PER^TM^ Nuclear and Cytoplasmic extraction Reagents (Pierce, Rockford, IL); following the manufacturer’s protocol. 200 ~300 mg frozen tissue was crushed into powder in liquid nitrogen before adding the extraction reagent. A thick fat layer from mammary gland tissue was removed after centrifugation before collecting the cytoplasmic extract. 50 μg of nuclear extract were electrophoresed on SDS-polyacrylamide gel and transferred onto Immobilon-FL membrane (Millipore, Billerica, MA); ECL Plus (GE Healthcare, Piscataway, NJ) were used to visualize protein bands. Other antibodies used were: ATF3 (Santa Cruz [Santa Cruz, CA]:C-19); Jun (Calbiochem [San Diego, CA]:OP55); Ccnd1 (Oncogene [San Diego, CA]:CC12).

Chromatin immunoprecipitation (ChIP)

ChIP assays were performed using the SimpleChipTM Enzymatic Chromatin IP Kit (Cell Signaling #9003) essentially as described by the manufacturer. Four 100 mm dishes of EMT6 cells (~1x 10^7^ per dish) were cross-linked with 1% formaldehyde (final concentration) at room temperature for 10 min; reactions were stopped by adding glycine. Cross-linked chromatin was digested with micrococcal nuclease, and then sonicated (Fisher Scientific Sonic 550, at power setting “3”, 3 x 20 s) to break the nuclear membrane. This produced DNA with a size distribution of approximately 150-900 bp, as determined by electrophoresis. For each immunoprecipitation, 100ul (~13ug) DNA was incubated overnight at 4°C with either 1ug normal rabbit IgG (Cell Signaling, # 2729 from kit) as negative control, or with 2 ug of specific antibody: ATF3 (Santa Cruz, sc-188) or Jun (Santa Cruz, sc-45). DNA present in the immunoprecipitate was purified as described by the manufacturer.

PCR reactions specific for the genes of interest were carried out with DNA purified from each immunoprecipitate and analyzed by agarose electrophoresis. Positive control lanes utilized 2% of the input DNA. Gene regions containing putative AP-1 sites as described in the text analyzed were as follows; numbering is relative to the transcriptional start site as given in the ENSEMBL database: Jun (-5042 to -4876); Tcf7 (-3717 to -3575 and -2778 to -2557); Wnt7b (-4860 to -4681 and -1035 to -852); Snai2 (-5027 to -4835); Snai1 -1465 to -1240). As a non-specific control, a region was analyzed upstream of Jun (-6359 to -5979) that contained no sequences closely related to the AP-1 sites.
