# Supplementary figures and images for: Activation of the Canonical Wnt/β-Catenin Pathway in ATF3-Induced Mammary Tumors
Source: PLoS One. 2011 Jan 31;6(1):e16515. doi: 10.1371/journal.pone.0016515 (PMC3031586; doi:10.1371/journal.pone.0016515)

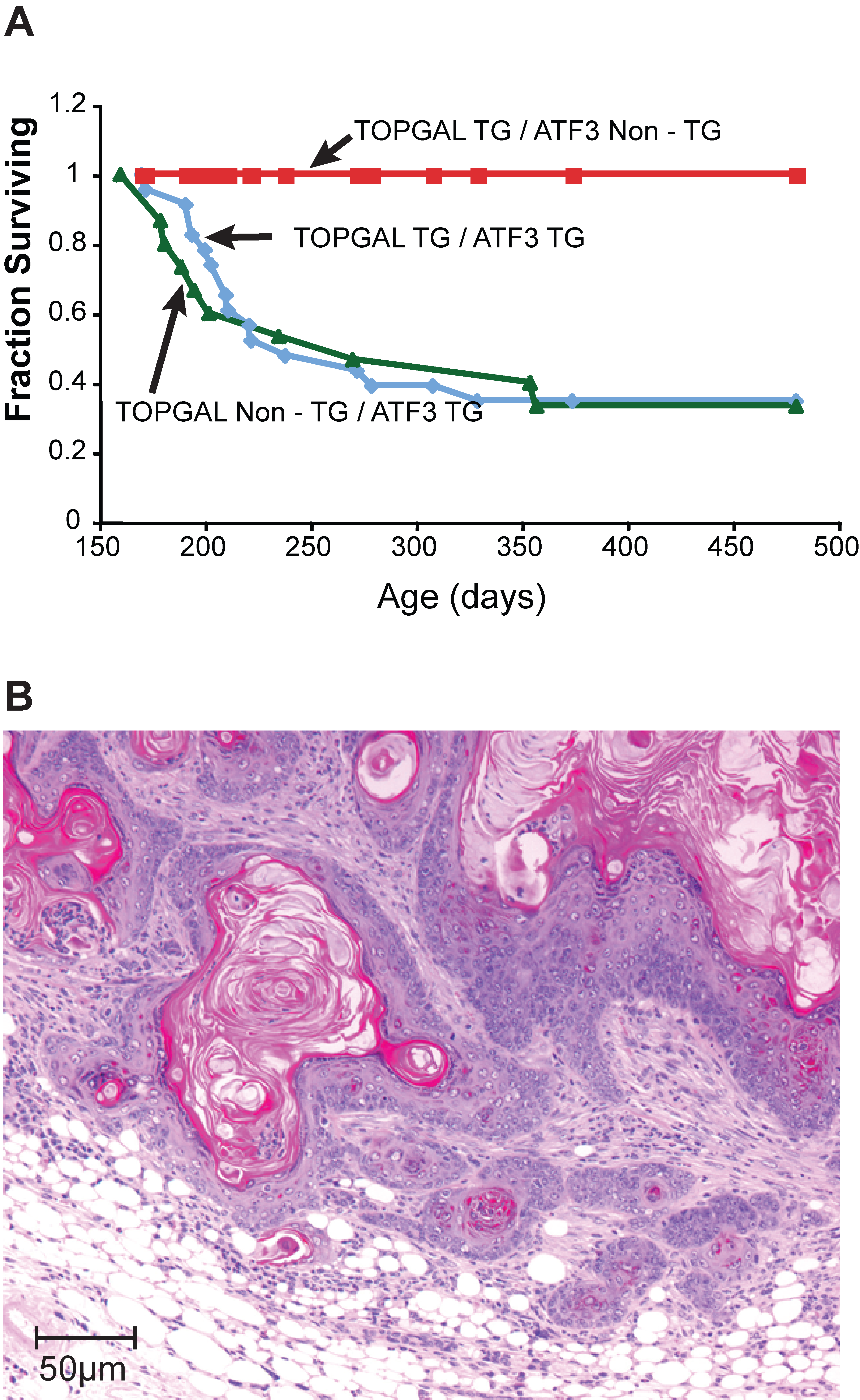

Supplement: Figure S1 — Mammary tumorigenesis in parous BK5.ATF3; TOPGAL mice. Singly transgenic animals from the BK5.ATF3 line and the TOPGAL line were mated to produce double heterozygotes (ATF3+/−, TOPGAL+/−). The doubly heterozygous females and their singly transgenic (ATF3−/−, TOPGAL+/−) littermates were allowed to mate and raise litters twice, and then monitored for mammary tumor formation until 16 months of age. Tumor-bearing animals were sacrificed when a tumor reached 1.5 cm in its longest dimension. A. Survival curves are shown for several different genotypes. Data for TOPGAL non-TG/ATF3 TG mice is from reference (Wang et al., 2008). B. Tumors arising in (ATF3+/−, TOPGAL+/−) females were harvested and analyzed by histopathology. Scale bar in panel B = 50 µm. (TIF) [file pone.0016515.s002.tif]
